# Analysis of human satellite cell dynamics on cultured adult skeletal muscle myofibers

**DOI:** 10.1186/s13395-020-00256-z

**Published:** 2021-01-04

**Authors:** Peter Feige, Eve C. Tsai, Michael A. Rudnicki

**Affiliations:** 1 Sprott Center for Stem Cell Research, Regenerative Medicine Program, Ottawa Hospital Research Institute, 501 Smyth Road, Ottawa, ON K1H 8L6 Canada; 2 Department of Cellular and Molecular Medicine, Faculty of Medicine, University of Ottawa, Ottawa, ON Canada; 3 Department of Medicine, Faculty of Medicine, University of Ottawa, Ottawa, ON Canada; 4 Department of Surgery, Division of Neurosurgery, Faculty of Medicine, University of Ottawa, Ottawa, ON Canada; 5 Ottawa Hospital Research Institute, Neuroscience Program, Ottawa, ON Canada

**Keywords:** Human skeletal muscle, Satellite cell, Muscle stem cell, Myofiber culture

## Abstract

**Background:**

Maintaining stem cells in physiologically relevant states is necessary to understand cell and context-specific signalling paradigms and to understand complex interfaces between cells in situ. Understanding human stem cell function is largely based on tissue biopsies, cell culture, and transplantation into model organisms.

**Methods:**

Here, we describe a method to isolate post-mortem intact human muscle myofibers and culture muscle stem cells within the niche microenvironment to assay cellular dynamics, stem cell identity, stem cell hierarchy, and differentiation potential.

**Results:**

We show human myofiber culture maintains complex cell-cell contacts and extracellular niche composition during culture. Human satellite cells can be cultured at least 8 days, which represents a timepoint of activation, differentiation, and de novo human myofiber formation. We demonstrate that adult human muscle stem cells undergo apicobasal and planar cell divisions and express polarized dystrophin and EGFR. Furthermore, we validate that stimulation of the EGFR pathway stimulates the generation of myogenic progenitors and myogenic differentiation.

**Conclusions:**

This method provides proof of principle evidence for the use of human muscle to evaluate satellite cell dynamics and has applications in pre-clinical evaluation of therapeutics targeting muscle repair.

**Supplementary Information:**

The online version contains supplementary material available at 10.1186/s13395-020-00256-z.

## Background

Skeletal muscle is a complex tissue, responsible for mobility, thermoregulation, and breathing. Skeletal muscle maintenance, growth, and repair are facilitated by muscle resident stem cells (satellite cells) which reside within skeletal muscle, resting within a specialized cleft underneath the basal lamina [1]. Muscle homeostasis requires a balance between satellite cell self-renewal and differentiation to facilitate efficient repair over time and in response to injury [2]. Changes to satellite cell-intrinsic signalling, satellite cell-niche interactions, or the regenerative context in conditions such as ageing or muscular dystrophy alter the kinetics of muscle repair (reviewed in [2]). Improved modeling of human satellite cell dynamics in a physiologically relevant context will improve our understanding of signalling pathways pertinent to muscle repair in humans.

With age, progressive loss of satellite cell number is observed with functional decline in skeletal muscle [3, 4]. In aged mice, extrinsic changes to soluble ligands in the niche [5–7], increased fibrosis [8], and intrinsic changes in satellite cells such as constitutively active p38α/β signalling, or elevated JAK-STAT signalling alter cell fate, reduces regenerative capacity in muscle [9–12] and biases aged satellite cells to asymmetric modes of division producing committed progenitors and exhausting the muscle stem cell pool [10]. Chemical and physical cues present in young and healthy muscle act to balance satellite cell quiescence, self-renewal, and asymmetric division to maintain the satellite cell pool in a state amenable to rapid activation in response to injury [9, 10, 13]. Understanding if these processes are conserved or altered in human satellite cells is important to develop methods improving endogenous satellite cell-mediated repair.

Applying laboratory insights to human disease is failure-prone, where roughly 1 in 10 pharmaceuticals entering clinical trials gain approval by the FDA [14] with efficacy being a major concern [15]. The value of animal models for predicting clinical responses to therapy remains controversial and partially explained by publication biases, methodological flaws in animal experiments, non-transparent data reporting, and fundamental differences in human and animal physiology limiting generalizability of results [16, 17].

Improved methods investigating satellite cell biology such as satellite cell isolation [18], satellite cell transplantation [19, 20], and myofiber culture [21] have improved our understanding of satellite cell heterogeneity [22–24], hierarchy [24–26], regenerative capacity [10, 27, 28], and the therapeutic potential of augmenting muscle stem cell repair [23, 29, 30].

Development of novel 3D culture systems [31], bioengineering approaches modeling human muscle [32, 33], isolation of primary muscle cells from human cadavers [34], humanized mouse models of muscular dystrophy [35], and advancements in iPSC-derived myogenic cells [36, 37] have uncovered unique aspects of human muscle disease; however, no system robustly recapitulates the complexity in the adult human myofiber chemical composition, physical composition, or cellular composition [33]. In model organisms, culturing myofibers harboring satellite cells maintains extracellular matrix composition [1], myofiber rigidity [38], and endogenous niche interactions [22] to provide a more relevant ex vivo culture paradigm [2].

Understanding the satellite cell-intrinsic changes occurring with disease provides important insight into treatments addressing the etiology of muscle disease. Duchenne’s muscular dystrophy (DMD) is a devasting lethal disease where loss of dystrophin leads to severe deficit in myofiber structural integrity [39]. Our lab made the seminal discovery that in mice, muscle stem cells also express dystrophin where it is localized together with the dystrophin-associated glycoprotein complex at the basal cortex of a portion of dividing cells against the basal lamina. Dystrophin recruits Par1b/Mark2 to establish polarity that is required for asymmetric apicobasal cell divisions. In the absence of dystrophin, loss of polarity leads to a significant reduction in asymmetric divisions and reduced generation of progenitors. Consequently, regeneration is impaired contributing to disease progression [40]. Dystrophin expression has been noted in dispersed fetal human myogenic cells [41], but has yet to been observed in adult muscle stem cells in situ.

Dystrophin-mediated signalling influencing satellite cell polarity is one mechanism promoting myogenic progenitor formation. We have previously shown that activation of the EGFR pathway can stimulate the formation of apicobasal-oriented mitotic centrosomes in a dystrophin-independent manner in dystrophin-deficient mice [23]. Mechanistically, polarized phosphorylated EGFR can recruit the mitotic centrosome regulator Aurora Kinase A to facilitate apicobasal-oriented mitotic divisions and the formation of myogenic progeny through asymmetric divisions. Both dystrophin and EGFR-mediated satellite cell polarity are influenced by the myofiber 3D microenvironment, where apicobasal-oriented satellite cell divisions can be observed myofiber culture in situ and in vivo in response to injury [22, 42]. Whether this pathway similarly functions in human muscle stem cells has remained an unanswered question.

Myofiber culture [21] has led to multiple seminal discoveries in mouse satellite cell biology [10, 22, 23, 28, 40]. We hypothesized human myofiber isolation could be feasible and provide insight into fundamental differences in human and mouse satellite cell biology. Common methods of isolating human muscle such as punch biopsy are not suitable to culture as sarcolemma membrane damage results in calcium overload and myofiber hyper contraction [43]. Maintaining healthy myofibers amenable for culture requires tendon to tendon isolation followed by enzymatic digestion of the extracellular matrix to release single muscle fibers [21], where healthy non-contracted myofibers are cultured to maintain endogenous niche interactions.

Here, we report that by utilizing primary human myofibers, we can model human muscle stem cell dynamics in a chemically, physically, and cellularly relevant context. This method is amendable to model human muscle resident stem cells including satellite cells and in the future feasibly other resident cell types in muscle such as fibroadipogenic precursors, mesenchymal cells, fibroblast, pericyte, endothelial cells, and tenocytes [44, 45]. Myofibers can be prepared within 3 h of surgical excision and can be cultured ex vivo for at least 8 days which reflects satellite cell expansion, differentiation, and de novo human myofiber formation. The ability to directly assay genetic pathways in human satellite cells in a relevant context provides an exciting opportunity for pre-clinical testing and to develop causative relationships in human satellite cell signalling.

We show adult human satellite cells undergo apicobasal and planar cell divisions and express polarized dystrophin and EGFR. Moreover, we report that stimulation of the EGFR pathway augments satellite cell generation of myogenic progenitors. Therefore, this method holds the potential to accelerate therapeutic development by evaluating genetic pathways relevant to human muscle disease.

## Methods

### Experimental subjects

This study was approved by the Ottawa Hospital Research Ethics Board and informed consent was obtained prior to proceeding. Samples were harvested from patients that had already been consented for organ donation through the Trillium Gift of Life Network following neurological determination of death in compliance with guidelines from The Ottawa Hospital Research Ethics Board. Donors did not present with cachexia or muscular dystrophy by independent chart review.

### Histological analysis of muscle cross sections

Minimum fiber Feret and myofiber surface area measurements were performed using the semi-automated SMASH software plugin for MATLAB 2015a described previously [46]. Total myofiber count per cross section was verified by manual validation of SMASH myofiber masks and original images. Myofiber types were counted manually across each cross section studied.

### Human *Psoas* minor isolation

**Fig. 1:**
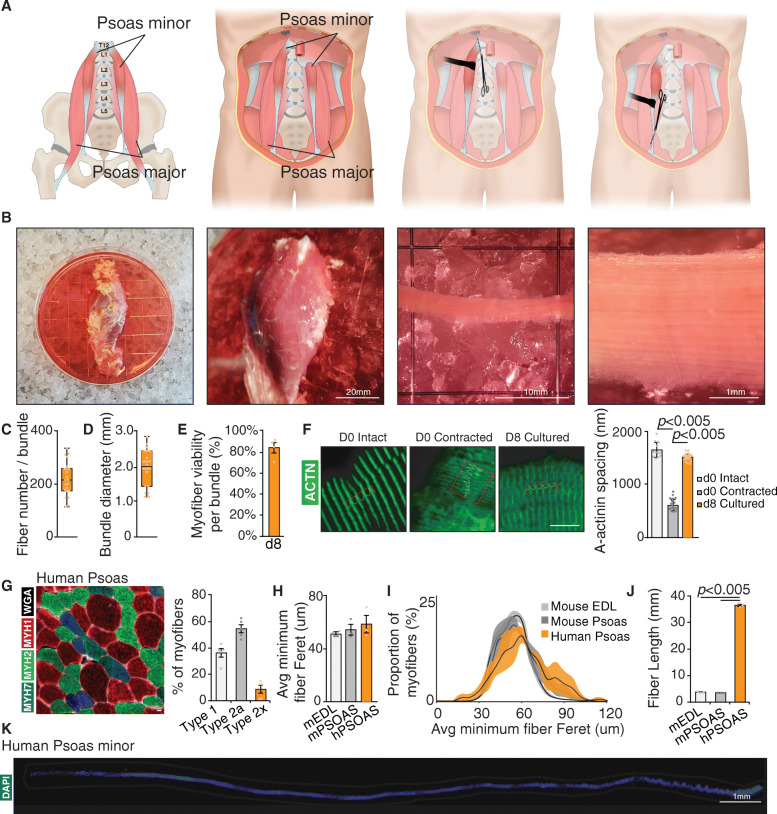
Human *Psoas* minor muscle is amenable for myofiber culture. **a** Graphic overview of anatomical dissection of *Psoas* minor muscle from organ donors. **b** Photographic overview of myofiber bundle isolation from surgically removed human *Psoas* minor muscle. Quantification of **c** single myofibers per bundle and **d** myofiber bundle diameter, whiskers represent min and max values. **e** Myofiber viability as quantified by presence of hypercontracted myofibers. **f** Representative images and quantification of myofiber sarcomere spacing from intact, contracted, and cultured myofibers stained with α-Actinin (green). **g** Representative image and quantification of human *Psoas* fiber type stained for type I myofibers (blue), type IIa myofibers (green), type IIx myofibers (red), and wheat germ agglutinin (White). **h** Quantification of average minimum fiber Feret and **i** minimum myofiber Feret proportion of myofibers from human *Psoas* myofibers compared to mouse *Extensor digitorum longus* and mouse *Psoas* muscles using SMASH software. **j** Quantification of human *Psoas* myofiber length compared to mouse *Extensor digitorum longus* and mouse *Psoas* muscles. **k** Representative image of a single isolated human *Psoas* minor myofiber stained with DAPI (blue). **c**, **d**, **f** Error bars represent means ± SD, **e**, **g**–**j** Error bars represent means ± SEM; *p* values are listed. **c**, **d** *n* = 22 myofiber bundles, **e** *n* = 4 biological replicates, **f** *n* = averages from 12 to 19 myofibers per condition, **g** *n* = 5 biological replicates. **h**–**j** *n* = 3 biological replicates

Immediately after the organ retrieval process was completed, the psoas minor muscle was exposed using Deaver retractors to visualize tendinous insertions into the iliopectineal arch. A graphic overview of the procedure is provided in Fig. 1a. The distal aspect of the psoas tendon was cut, and the psoas dissected to its origin on the vertebral bodies and cut immediately adjacent to the vertebra. It is critical to minimize tension on the *Psoas* minor during dissection. The free *Psoas* minor was the immediately placed into sterile, cold transport media (DMEM, 110 mg/ml Pyruvate), and maintained on ice during transport.

### Human myofiber preparation

In a sterile environment, *Psoas* muscle is placed in an appropriate vessel buffered with ice and containing enough transport media to submerge. A photographic overview of the procedure is presented in Fig. 1a and S1A. Using a dissecting microscope and blunt micro scissors, adipose tissue, and free epimysium is dissected to visualize muscle fascicle tendon-tendon organization. Using blunt tipped micro scissors and blunt tipped tweezers, fascicle boundaries are gently retracted and perimysium is cut to free fascicle bundles continuing along the fascicle boundaries without freeing bundles from the tendon. Fascicles are further dissociated to bundles containing ~ 200 muscle fibers by measuring with a precision micro ruler (TDI) to ensure ~ 2 mm in diameter bundles are prepared. Using sharp micro scissors, tendons are cut to release free myofiber bundles avoiding injury to myofibers. Myofiber bundles are then placed in excess filtered myofiber culture media (DMEM, 110 mg Pyruvate, 20% FBS, 2.5ng/ml bFGF, 1% Chicken Embryo extract–25 ml/bundle) and cultured at 37 °C in normoxia. For EGF treatment, human recombinant EGF was supplemented to the media at isolation at 100 ng/mL where 1% BSA in PBS served as vehicle control. Media is changed every day. Following culture, myofibers are fixed by placement in excess warmed 4% paraformaldehyde for 5 min where excess 4% paraformaldehyde is then injected directly within myofiber bundles using an insulin syringe. Bundles are fixed for 30 min in 4% paraformaldehyde. Bundles are then moved to 0.4% paraformaldehyde for 12 h followed by extensive washing. Samples are maintained in PBS containing ProClin950 at 4 °C for long-term storage. For analysis of full-length bundles, individual myofibers may be isolated by retraction using blunt tipped tweezers along the length of a myofiber bundle. For cross-sectional analysis of cultured bundles, bundles are frozen whole prior to fixing or fixed bundles are segmented, hydrated for 12 h in a sucrose gradient and frozen by imbedding in OCT and freezing by nitrogen cooled isopentane. For bundle analysis, fixed cultured myofiber bundles are manually dissociated to 5–15 myofiber bundles and segmented to length amendable to microscopic analysis and to improve antibody penetration.

### Immunostaining and antibodies

Myofibers and myofiber bundles are processed by identical means. Fixed myofiber samples are washed in PBS and permeabilized in 0.4% Triton-X100 for 1 h with rocking. Samples are washed in PBS containing 125 mM glycine 3× in excess buffer until no appearance of detergent remains. Samples are blocked using TrueBlack as per manufacturer’s instructions. Samples are then blocked in blocking buffer containing 5% normal donkey serum for 3 h at room temperature to overnight at 4 °C with rocking. Samples are washed in PBS and primary antibodies are applied for 24 h at room temperature with rocking. Samples are washed in excess PBS 5× 30 min each with rocking. Secondary antibodies are applied 24 h at room temperature with rocking in the dark. Samples are washed in excess PBS for 30 min with rocking and DAPI is applied for 1 h with rocking. Samples are washed in excess PBS 5× 30 min each with rocking in the dark. Samples are then passed through a serial 20–80% glycerol series and mounted onto slides in glycerol mounting media containing 0.1 M n-propyl gallate.

Antibodies used in the study are as follows: Mouse anti-Pax7 (1:2, DSHB, Cat. no. Ab528428; RRID: AB_528428), rat anti-Laminin (1:1000, Sigma, Cat. no. L0663; RRID: AB_477153), Mouse anti-Dystrophin (1:500, DSHB, Cat. no. MANEX1011B; RRID:AB_1157876), Rabbit anti-p-EGFR (1:250, Cell Signaling technology, Cat. no. 3777S; RRID: AB_2096270), Mouse anti-M-Cadherin (1:500, BD Biosciences Cat. no. 611101, RRID:AB_398414), Rabbit anti-MyoD (1:500, Abcam, Cat. no, ab133627), Mouse anti-Syndecan-4 (1:500, Santa Cruz Biotechnology Cat. no. sc-12766; RRID:AB_628314), Rabbit anti-Annexin-5 (1:500, Abcam, Cat. no. ab14196, RRID:AB_300979), Mouse anti-MyoG (1:500, Novus, Cat. no. MAB66861; RRID:AB_10973343), Mouse anti-α-Actinin (1:1000, Sigma, Cat. no. A7732; RRID:AB_2221571), Mouse anti-MHC (1:1000, DSHB, Cat. no. MF 20; AB_2147781), Mouse anti-MyH2 (1:100, DSHB, Cat. no. SC-71; AB_2147165), Mouse anti-MyH1 (1:100, DSHB, Cat. no. 6H1; AB_2314830), Mouse anti-MyH4 (1:100, DSHB, Cat. no. BF-F3; RRID: AB_2266724), Mouse anti-MyH7 (1:100, DSHB, Cat. no. BA-F8; RRID: AB_10572253), Rat anti-Perlecan (1:500, NSU Bioreagents, Cat. no. V2600; RRID:AB_2119238), Rabbit anti-Ki67 (1:1000, Abcam, Cat. no. ab15580; RRID:AB_443209), and Wheat Germ Agglutinin Alexa 488 conjugate (1:1000, Fisher, Cat. no. W11261).

### Scanning electron microscopy

Samples were washed with water prior to dehydration in a 35–100% graded series of ethanol. Samples were critical point dried in dry 100% ethanol using liquid CO2 as transition fluid. CO2 was exchanged at 5-min intervals for 8 rounds followed by a final 3 h release. Samples were maintained under dust-free desiccation following critical point drying. Samples were mounted on aluminum stages using double-sided carbon tape. Mounted samples were sputter coated with gold for 1 min (~ 10 nm) and stored under dust-free desiccation prior to imaging on a phenom Pro-X scanning electron microscope at an accelerating voltage of 15 kV.

### Imaging and analysis

Human *Psoas* myofiber bundles were analyzed on a Leica TCS SP8 confocal microscope equipped with HC PL APO 20× IMM CORR objective with HyD and PMT detectors. Filters and detectors were set to maximal bandwidth and sensitivity to limit bleed through between channels. Tile scans were stitched directly following acquisition using the Leica LAS AF software. Manual image analysis was performed using ImageJ and presented images represent maximum intensity projections unless stated otherwise. Myofibers partially encompassed in Z-stacks were excluded form analysis.

### Quantification and statistical analysis

Compiled data are expressed as mean ± standard deviation (SD) or mean ± standard error of the mean (SEM) as stated. Experiments were performed with a minimum of three biological replicates unless stated otherwise. For statistical comparisons of two conditions, the Student’s *t* test was used. Data is presented as paired (mean (control vs treatment)) for direct comparison of biologically matched samples and unpaired (mean control vs mean treatment) where appropriate. Statistical testing was performed for each histogram where appropriate and was reported where significant. *p* values are provided for each statistical test performed. No data were removed as outliers. Experimental design incorporated user blinding when possible. Statistical analysis was performed in GraphPad Prism or Microsoft Excel.

### Key resources

Key resources are listed in Table S2.

## Results

### Human *Psoas* muscle is amenable for satellite cell culture in situ

To evaluate our hypothesis that human myofibers could be cultured in a laboratory setting, we decided to isolate primary human tissue from neurological determination of death organ donors following ethics approval and informed consent. We evaluated muscle groups for suitability in myofiber culture where surgical access to both tendinous insertions is feasible and where myofibers are short to facilitate manipulation in a laboratory setting with common plasticware and reasonable reagent volumes. We identified the small muscles of the hand, including the *Abductor* or *Flexor Pollicis Brevis*, the *Flexor Digitorum Brevis* of the foot or the *Pronator Quadratus* of the forearm would be ideal candidates due to their size and accessibility; however, clinical availability of these muscles limited laboratory testing. We reasoned that an alternative strategy would involve isolating muscle with large angles of pennation, where individual myofibers are aligned at an angle oblique to the longitudinal angle of muscle contraction. These muscles provide a mechanical advantage over shorter contraction distances and possess shorter myofiber fascicles attaching to fibrous aponeuroses running along the periphery of the muscle [47]. These muscle types would also allow myofiber isolation from partial muscle dissections.

We identified the human *Psoas* muscle as a moderately pennate muscle attached to the T12 vertebrae and the Lacunar ligament (Fig. 1a) with some variable presence within populations [48, 49], as a good candidate to isolate myofibers. We obtained post-mortem muscle samples from neurologically determined deceased organ donors (2♀, 1♂) with a mean age of 61 ± 9.4 years (range 50–68) (Table S1). Donors did not have muscular dystrophy and did not exhibit muscle cachexia. Muscle samples were 24.35 ± 13.56 g in mass and 11.42 ± 2.15 cm in length and isolated within 2–4 h from cardiac death.

Mouse myofiber isolation requires enzymatic digestion of the extracellular components from isolated mouse *Extensor digitorum longus* muscle to release single myofibers amenable for culture. To test if human *Psoas* myofibers could be prepared similarly, we subjected myofiber isolated *Psoas* biopsies to collagenase digestion. Isolated *Psoas* muscle was resistant to digestion using collagenase type 1, type 2, type 3, type 4, type 5, type 6, type 7, or elastase buffers due to the thick perimysium, endomysium, extracellular matrix (ECM), and vascular networks present (data not shown). Thus, the established methods of myofiber isolation used in mouse studies are not appropriate for human myofiber isolation.

Therefore, we manually dissected myofiber bundles from *Psoas* samples from tendon to tendon (Figure S1A). Manually dissected myofibril bundles contained 220 ± 63 myofibers (Fig. 1c) and were 1.9 ± 0.5 mm in diameter (Fig. 1d). Myofiber bundles displayed heterogeneous viability, where excess tension during surgical excision or processing resulted in samples containing hyper contracted myofibers which were excluded from further analysis (Figure S1A). Successful myofiber preparations contained intact myofibers where 83 ± 5% of myofibers within a bundle did not exhibit any signs of hyper contraction or injury (Fig. 1e). Injured myofibers can be distinguished into hypercontracted myofibers, moderately injured and minor injured subgroups. Hypercontracted myofibers (Figure S1B) show bisected myofiber segmentation within an extracellular matrix scaffold while myofibers with moderate damage (Figure S1C) exhibit widespread disorganization of sarcomeric banding and significant autofluorescence. Myofibers with minor damage (Figure S1D) exhibit focal autofluorescence and invagination of the extracellular matrix and maintain myofiber-cell-ECM contact. Quantification of sarcomere spacing through α-Actinin staining (Fig. 1f, S1E-F) showed significantly shorter sarcomere banding in contracted myofibers, whereas healthy myofibers can be maintained in culture for at least 8 days without myofiber contraction or loss of sarcomere disorganization. Together, this data suggests that human *Psoas* myofiber bundles can be isolated and maintained in culture without consequence to myofiber integrity.

Mouse and human myofibers exhibit different histological characteristics, where human skeletal muscle generally lack glycolytic myosin heavy chain type IIb (MyHC IIb) muscle fibers in favor of type IIx [50]. Additionally, myofiber type has an impact on satellite cell response to exercise, where satellite cells resident to oxidative MyHC type I human muscles show augmented expansion following aerobic training [51]. We hypothesized that myofiber type would influence satellite cell fate in human *Psoas* myofiber cultures and could confound translating signalling pathways identified in mice to human satellite cells.

We evaluated myofiber type and histological profiles of human *Psoas* myofiber bundles, mouse *Extensor digitorum longus* (EDL) and mouse *Psoas* myofibers to better correlate difference in rodent and human satellite cell biology. Isolated human *Psoas* myofiber bundles were composed of mixed 36.5 ± 3.1% slow (type I) and 63.5 ± 6% fast (type IIa, IIx) myofibers compared to 14.4 ± 2% type IIa and 62.5 ± 1.2% type IIb fast twitch mouse *Psoas* or 11 ± 1.2% type IIa and 72.9 ± 0.7% type IIb EDL muscles (Figure S1G). Human *Psoas* myofibers do not significantly differ in minimum fiber Feret (Fig. 1h) or myofiber surface area (Figure S1I) compared to mouse *Psoas* or EDL fibers where a subset of human *Psoas* myofibers are hypertrophic (Fig. 1I, S1J). Human myofibers however are roughly 10-fold longer than mouse *Psoas* or EDL myofibers (Fig. 1j–k) (36.4 ± 0.4 mm versus 3.34 ± 0.05 mm and 3.60 ± 0.12 mm). Taken together, this data suggests that human *Psoas* myofibers display distinct histological characteristics from mouse *Psoas* or EDL muscle likely due to requirements for hip flexion and posture in an erect position.

### Human satellite cells expand in myofiber culture

Satellite cells remain mitotically quiescent but are poised to activate and enter the cell cycle in response to extrinsic cues such as exercise or trauma [2]. In rodents, this can be achieved by experimental models of injury, muscle digestion for stem cell isolation [52, 53] or in the case of myofiber preparation, digestion with collagenase and exposure to growth factors in cell culture [21]. To evaluate if human satellite cells from *Psoas* myofiber cultures spontaneously activate in vitro, we evaluated basal satellite cell numbers immediately following surgical excision and during culture where we analyzed an average 1.8 ± 1.1 mm length of myofibers per experiment (Figure S2A).

**Fig. 2:**
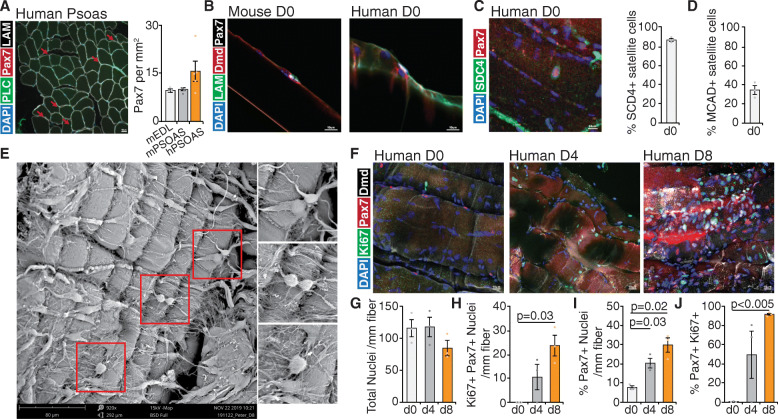
Human satellite cells expand in culture. **a** Representative image and quantification of satellite cell density from human *Psoas* minor muscle cross sections stained with DAPI (blue), Perlecan (green), Pax7 (red), and Laminin (white). **b** Representative images of mouse and human satellite cells in the niche at isolation stained for DAPI (blue), Laminin (green), dystrophin (red) and Pax7 (white). **c** Representative image and quantification of human satellite cells expressing Syndecan-4 (SDC4) at isolation stained with DAPI (blue), Syndecan-4 (green), and Pax7 (red). **d** Quantification of human satellite cells expressing M-Cadherin (MCAD) at isolation. **e** Representative scanning electron micrograph of cultured human myofibers (day 8) showing maintenance of myofiber extracellular matrix composition and cell-cell contacts. **f** Representative images of human myofibers in culture from day 0, day 4, and day 8 stained with DAPI (blue), Ki67 (green), Pax7 (red), and dystrophin (white). Quantification of **g** total nuclei per mm of myofiber, **h** satellite cells expressing Ki67 per millimeter of myofiber, **i** percentage of satellite cells per total nuclei per mm of myofiber, **j** proportion of satellite cells expressing Ki67 on myofibers. **a**, **c**, **d**, **g**–**j** Error bars represent mean ± SEM; **a** *n* = 3 biological replicates for mouse EDL, mouse *Psoas*, *n* = 5 biological replicates from human *Psoas*. **c**, **d**, **g**–**j** *n* = 3 biological replicates

Human *Psoas* muscle has generally more satellite cells per mm^2^ (15.7 ± 3.0) compared to mouse *Psoas* (9.5 ± 0.5) or EDL myofiber cross-sections (10.0 ± 0.36) following immunofluorescence staining for Pax7 (Fig. 2a). We observed by immunofluorescence staining for Pax7 that human *Psoas* myofibers with centrally located nuclei possess significantly increased (75.1%, 8.67 ± 1.6 versus 15.2 ± 3.1) satellite cells per millimeter of myofiber (Figure S2B-C) possibly due to prior repair in vivo.

Human *Psoas* satellite cells reside within the niche at isolation (Fig. 2b), where 86.5 ± 5.25% of human satellite cells express the cell surface marker Syndecan-4 (Fig. 2c, S2D, F) and 34.8 ± 7.6% heterogeneously express M-Cadherin (Fig. 2d, S2G). Most satellite cells maintain Syndecan-4 expression in culture (84.1.0 ± 6.7%) (Figure S2F) and do not express the apoptotic marker Annexin-5 (Figure S2D).

To evaluate myofiber integrity and maintenance of cell-cell and ECM interactions throughout culture, myofiber bundles were subject to scanning electron microscopy. Electron micrographs (Fig. 2e) show myofiber bundles exhibit no apparent tearing or porosity and maintain complex cell-cell and cell-ECM interactions following 8 days in culture. This data suggests human satellite cells expand locally in response to prior trauma and express and maintain Syndecan-4 expression throughout culture.

To directly assess satellite cell expansion in culture, we quantified satellite cell numbers throughout culture and double labelled with Pax7 and Ki67 to assess human satellite cell proliferation. We observed no statistical change in total nuclei per millimeter of myofiber following 8 days in culture (Fig. 2g) with a concomitant increase in proliferating satellite cells (0.017 ± 0.02 versus 23.8 ± 4.3 per millimeter myofiber day 8) (Fig. 2h) and other cell types (0.7 ± 0.2 versus 7.6 ± 0.8 per millimeter myofiber day 8) (Figure S2I) while the number of Ki67-satellite cell falls (9.18 ± 1.9 versus 2.06 ± 0.74 per millimeter myofiber day 8). Following 8 days in culture, human satellite cells make up 30.0 ± 3.7% of total nuclei along myofibers (Fig. 2I). As the number of activated satellite cells heterogeneously increases at day 4 (Fig. 2h, S2I), the first satellite cell divisions likely occur between day 3 and day 4 in culture (Fig. 2h, i), where the biological variance between samples is greater than that between myofibers (Figure S2K). Following 8 days in culture, most satellite cells (91.8 ± 1.2%) express Ki67 (Fig. 2j). Taken together, this data suggests human satellite cells actively proliferate in culture along with other resident cell types however with slower activation kinetics than that of mouse satellite cells.

### EGF stimulation promotes myogenic progenitor formation in human *Psoas* myofiber culture

**Fig. 3:**
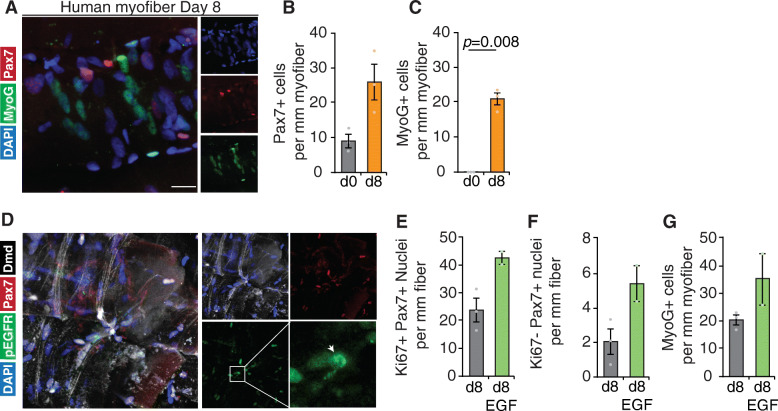
Human satellite cell expansion and differentiation can be tuned in situ. **a** Representative image (magnification from Figure S3A) of human satellite cells and Myogenin (MyoG) expressing differentiating progenitors cultured on 8-day human *Psoas minor* myofiber cultures stained with DAPI (blue), MyoG (green), and Pax7 (red). Quantification of **b** number of Pax7-expressing cells per millimeter of myofiber, **c** MyoG-expressing cells per millimeter of myofiber. **d** Representative images of human satellite cells expressing phosphorylated active EGFR (p-EGFR) stained with DAPI (blue), p-EGFR (green), Pax7 (red), and dystrophin (white). White arrow denotes localized p-EGFR expression. Quantification of **e** satellite cells expressing Ki67 per millimeter of myofiber and **f** Ki67-negative satellite cells per millimeter of myofiber following EGF treatment or vehicle control of human myofibers. **g** Quantification of number of MyoG-expressing cells per millimeter of myofiber following EGF treatment of human myofibers. **b**, **c**, **e**–**g** Error bars represent means ± SD (EGF) and means ± SEM (control); **b**, **c** *n* = 3 biological replicates. **d**–**g** *n* = 3 biological replicates control, *n* = 2 biological replicates EGF

To evaluate myogenic differentiation in *Psoas* myofiber culture, we examined myofibers by immunofluorescence staining for Myogenin (MyoG), a transcription factor expressed at the onset of differentiation [54]. Culturing *Psoas* myofibers for 8 days results in significant presence of MyoG-expressing cells (20.8 ± 1.8 per millimeter myofiber) concomitant with proliferation of satellite cells, where we do not observe co-labelling of Pax7 and MyoG by immunofluorescence (Fig. 3a–c, S3A). Additionally, by 8 days in culture, we can observe *de novo* myofiber formation occurring characterized by multiple organized and aligned myocytes expressing MyoG (Figure S3B-C) suggesting human *Psoas* myofiber culture may represent a paradigm to model human satellite cell activation, differentiation, and myofiber formation. This data indicates that by 8 days in culture, human *Psoas* satellite cells expand in number and express differentiation markers including MyoG.

We have previously established that mouse satellite cells express EGFR that is polarized before an asymmetric cell division to orient the satellite cell mitotic spindle in an apical-basal orientation [23]. To evaluate if human *Psoas* satellite cells integrate EGFR signalling, cultures were treated with EGF ligand throughout culture to stimulate EGFR activation. Following EGFR stimulation, myofibers were stained for the presence of phosphorylated EGFR, where most satellite cells express p-EGFR (Fig. 3d, Figure S3D), and its expression can become locally restricted as observed in activated mouse satellite cells [23]. This data suggests that the EGFR signalling pathway is activated following EGF treatment in human satellite cells in a manner analogous to mouse satellite cells.

To evaluate if EGFR augmented the production of myogenic progeny through the promotion of asymmetric division as found previously in mouse muscle [23], we examined fibers by immunofluorescence for Pax7, MyoG, and Ki67. Quantifying total nuclei per millimeter of fiber show no significant change following 8 days of EGF treatment (Figure S3H); however, treatment with EGF results in an appreciable increase in the number of proliferating satellite cells per millimeter of myofiber (56.7% paired, 78% unpaired, *n* = 3 control, *n* = 2 EGF) (Fig. 3e, S3F) and an increased proportion of satellite cells per myofiber (29% increase unpaired, 15% increase paired, *n* = 3 control, *n* = 2 EGF) (Figure S3G). Interestingly, EGF treatment increased the number of Ki67-negative satellite cells per millimeter of myofiber (161% increase unpaired, 118% increase paired, *n* = 3 control, *n* = 2 EGF) following 8 days in culture treated with EGF or vehicle control (Fig. 3f, S3H). However, the proportion of Pax7/Ki67 expressing cells was similar following EGF treatment (92% control vs 89% EGF) suggesting any effect as a mitogen was negligible. Concomitant with an increase in proliferating satellite cells, EGF treatment resulted in an appreciable increase in MyoG-expressing cells per millimeter of myofiber (69% increase unpaired, 78% increase paired *n* = 3 control, *n* = 2 EGF) (Fig. 3g, S3J). Therefore, we conclude that activation of EGFR signalling in muscle stem cells promotes the generation of progenitors, likely by stimulating asymmetric divisions, similarly between human and mouse.

### Human satellite cells undergo apicobasal and planar cell divisions and express polarized dystrophin

**Fig. 4:**
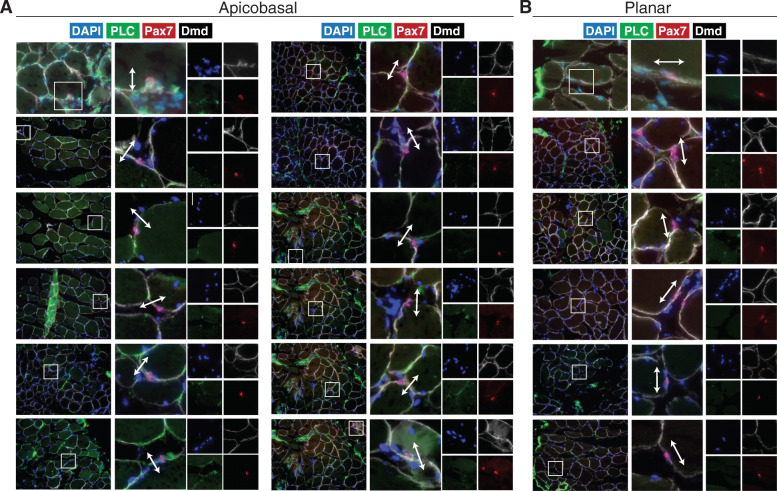
Human satellite cells can orient division angles. Representative images of **a** planar and **b** apicobasal-oriented human satellite cells in the niche at isolation stained with DAPI (blue), Perlecan (green), Pax7 (red), and dystrophin (white). *n* = 3 biological replicates

To evaluate the possibility of human satellite cells undergoing apical-basal-oriented asymmetric division, we examined myofibers by immunofluorescence staining throughout culture. Interestingly, we observed rare apicobasal and planar-oriented satellite cell doublets expressing Pax7 and residing in the niche from samples fixed at isolation and stained with Pax7, Perlecan, and Dystrophin (Fig. 4). As these cells are occupying the same niche space, we believe this is not due to random cell migration along the myofiber, suggesting that homeostatic repair mechanisms may undergo either mode of division.

**Fig. 5:**
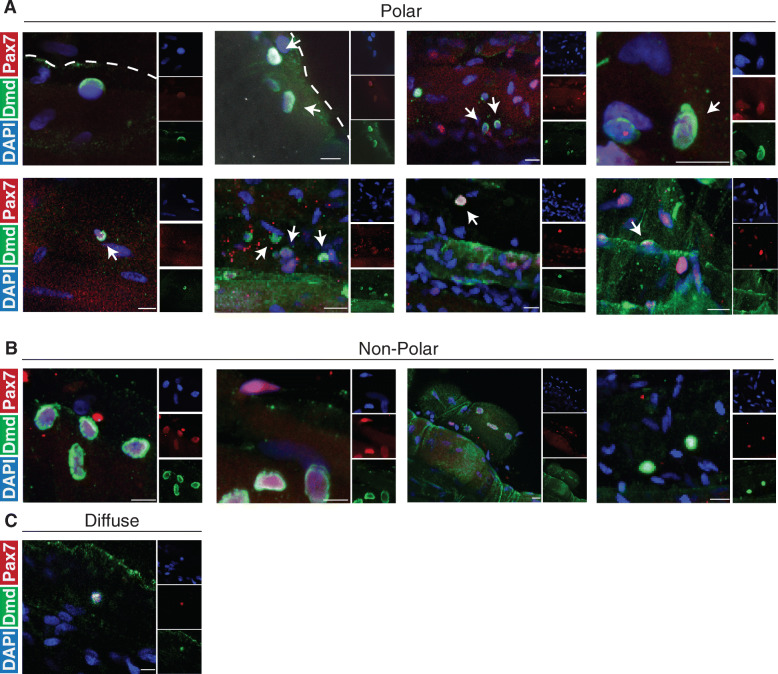
Human satellite cells express polarized dystrophin in culture. Representative image of human satellite cells cultured for four days and stained with DAPI (blue), Pax7 (red), and dystrophin (green) showing **a** polarized dystrophin localization, **b** non-polar dystrophin localization, and **c** diffuse dystrophin staining. *n* = 3 biological replicates

We additionally examined culture day 3 to day 4, a time point reflecting the first division in human satellite cells in culture. Strikingly, staining for the protein dystrophin (DMD), which can be polarized in mouse satellite cells to facilitate asymmetric division [40], shows strong expression and polarity on the basal surface of a subset of cultured satellite cells (Fig. 5a), along with a subset of satellite cells expressing non-polar dystrophin (Fig. 5b). We observe asynchronous expression of DMD in satellite cells along myofibers, where cells in close proximity can express DMD perhaps due to region-specific cues present within the myofiber microenvironment. This suggests that human satellite cells can polarize dystrophin to their basal surface interfacing with the extracellular matrix in an identical manner to activated mouse satellite cells.

## Discussion

**Fig. 6:**
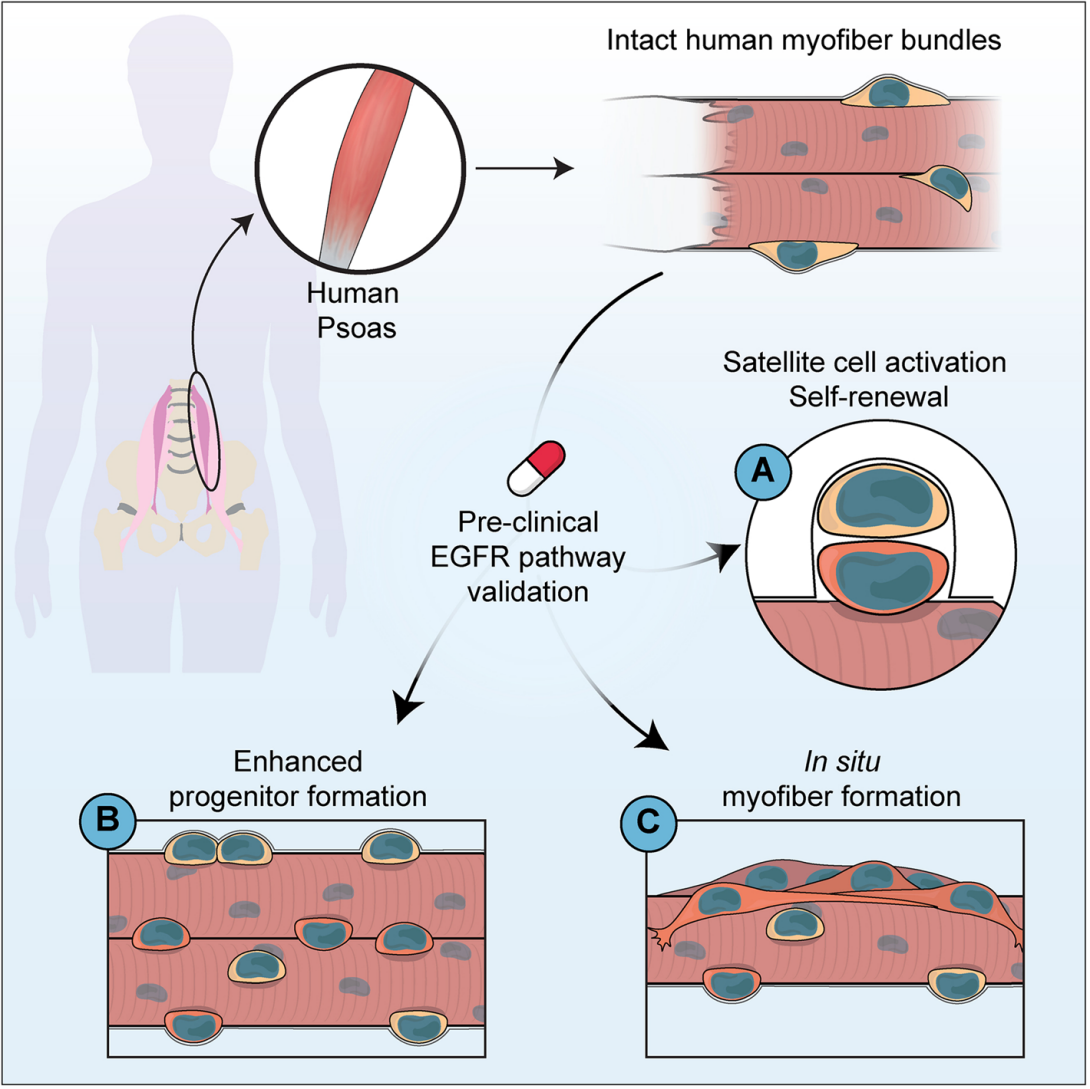
Graphical outline of human Psoas culture. Schematic diagram summarizing the procedure for the characterization of human satellite cell dynamics on cultured intact myofibers from the human Psoas muscle

Evaluating human stem cell dynamics in a relevant context is critical to model biological phenomena and generalize results to benefit human health. New methods to improve the pre-clinical evaluation of therapeutic strategies to augment endogenous stem cell activity hold promise to improve regenerative medicine outcomes in conditions such as Duchenne’s muscular dystrophy. Here, we developed a novel system to evaluate human satellite cell fate choices in a relevant context to interrogate human satellite cell biology and evaluate pre-clinical therapeutics in improving muscle regeneration (Fig. 6).

A balance in satellite cell proliferation, production of myogenic progeny, and return to quiescence is essential to maintaining muscle repair over a lifetime. Asymmetric stem cell division is one method to balance stem cell maintenance and the production of myogenic progeny, where following division one daughter cell maintains its stem cell state and one differentiates down the myogenic lineage. Asymmetric cell division is established through cells integrating extrinsic environmental cues to restrict cell fate determinants in a polarized manner such that when a cell orients its mitotic centrosomes parallel with internally polarized cell fate determinants, daughter cells will receive discrete cellular contents [2]. Typically, in mouse satellite cells, the daughter cell maintaining niche interactions with the basal lamina maintains its stem cell nature. Establishment of an apical-basal-oriented mitotic spindle is in part facilitated by the PAR polarity complex, where we have previously shown EGFR is spatially restricted before mitotic divisions to orient centrosomes through recruitment of Aurora kinase A and spindle assembly [23]. We hypothesized that as human *Psoas* myofibers maintain myofiber and extracellular matrix composition that human satellite cells in culture could integrate three-dimensional external cues to influence cell fate.

Our findings support that human *Psoas* myofiber cultures provide a new opportunity to culture human satellite cells to explore fate choices during satellite cell activation and differentiation. In our system, myofiber integrity is maintained (Fig. 1, S1) as well as cell polarity cues (Figs. 2b, e, 4, and 5) where treatment with EGF results in augmented production of myogenic progeny (Fig. 3e–g). As EGF treatment also increases the number of non-satellite cells expressing Ki67 (Figure S3I), it is possible that EGF influences cell survival or is acting as a mitogen on the varied cell populations within myofiber cultures. Further studies exploring other resident cell types such as fibroblast, pericyte, and fibroadipogenic precursor cells will better evaluate the specificity of EGF in culture. Additionally, studies exploring growth factor and oxygen penetration in myofiber bundles through staining of growth factors and incubation in oxygen sensing compounds such as Hypoxy Probe labels would further evaluate the potential effect of nutrient availability on heterogeneity in satellite cell activity within myofiber bundles. Studies exploring non-myogenic cell death occurring in culture day 4–8 may provide insight into the cellular dynamics along a myofiber during early muscle repair.

The observation that EGF treatment increases the number of non-proliferative satellite cells following treatment (Fig. 3f) suggests that the effect of EGF on satellite cells may not be acting as a general mitogen. Taken with an increase in proliferating satellite cells (Fig. 3e) and increased formation of myogenic progeny (Fig. 3g), supports the hypothesis that EGF treatment stimulates asymmetric division resulting in augmented production of non-cycling satellite stem cells (Pax7+Ki67−) and rapidly proliferating myogenic satellite cells similar to that observed in mouse [23]. Further studies are required to increase biological sample sizes to fully delineate the role of EGF on human satellite cells. Taken together, the human *Psoas* muscle provides an exciting tool to explore in niche satellite cell biology and facilitate translation of pre-clinical therapeutics from studies in model organisms to humans.

Previous studies have developed methods to assess human satellite cell expansion in vitro from primary tissue [55] through the culture of hypercontracted human myofiber fragments from punch biopsies. Myofiber fragments contained 1–8 bisected myofibers 2–3 mm in length and by 10 days in culture, 80% of nuclei were Pax7+ with a significant amount of Desmin expressing cells within the myofiber [55]. Transplantation of whole myofiber fragments resulted in limited engraftment into mouse recipients. Differences in timepoints and muscle groups assessed between our study and others [55] limit comparisons; however, in our hands’ injury to the myofiber through hyper contraction or bisection results in disorganized myofiber sarcomeres (Fig. 1f) and altered myofiber-ECM interactions (Figure S1B-C), where only minor focal damage is tolerated along myofibers to maintain myofiber-cell-ECM interactions (Figure S1D). Typically, hypercontracted fibers are discarded in experiments using mouse myofibers due to the abnormal behavior of satellite cells [56]. Additionally, cell polarity is maintained in *Psoas* myofiber culture (Figs. 3d and 5) and by 8 days ~ 30% of cells express Pax7 and ~ 20% represent committed progeny. Differences between the models could be attributed to differential activation cues, non-satellite cell survival, differential activation in different muscle groups, or limitations in our study including exogenous culture of myofibers, modest sample size, or differences in outbred human donors. This suggests human *Psoas* myofiber culture may reflect a model of homeostatic turnover or response to minor injuries such as load-induced trauma or de-innervation, while human myofiber fragments may represent a paradigm of rapid satellite cell activation in response to widespread myofiber damage.

Our findings provide proof-of-principle evidence to support that in addition to the mature myofiber, human satellite cells express polarized dystrophin during satellite cell activation and that human myofiber culture represents a novel paradigm to explore human satellite cell self-renewal and myogenic differentiation. We further validate that human satellite cells can undergo planar and apicobasal-oriented divisions and demonstrate the EGFR pathway to be of pre-clinical interest to augment the production of myogenic progeny through a conserved mechanism first discovered in mouse. Future studies with increased experimental size exploring the kinetics of cell cycle entry and quantification of division angles following the first satellite cell division (likely days 2–3) will shed important insight into the extent of symmetric and asymmetric satellite cell divisions occurring in human muscle.

## Conclusion

Human myofiber culture provides an exciting opportunity to evaluate pre-clinical drug efficacy in a relevant human context. This method provides an opportunity to assay satellite cell heterogeneity, stem cell potential, stem cell hierarchy, and activation kinetics with the potential to therapeutically interrogate pathways of interest. Additionally, this method provides an additional tool to validate phenomena observed in other model organisms, validate lead compounds for drug discovery and testing, reduce animal model use, and accelerate the evaluation of therapeutics improving human health. We envision this technique will aid in generalizing pre-clinical strategies into the clinical arena and may in the long term be appropriate as a personalized therapeutic tool.

## Supplementary Information

**Additional file 1:** Supplemental figures related to figures 1-3, patient information used in this study and key resource table. **Figure S1:** Myofibers from human Psoas muscle can be maintained in situ, Related to Fig. 1. A) Photographic overview of human Psoas minor myofiber bundle isolation showing expanded images of intact myofiber bundles (panel 9) and hypercontracted myofiber bundles (panel 10). Representative images of B) hypercontracted myofibers and C) myofibers with moderate damage stained for DAPI (Blue), α-Actinin (Green) and Myosin heavy chain (MF20, Red). D) Representative image of myofibers with minor damage stained for DAPI (Blue), Dystrophin (Green), Laminin (White) and IgG (Red). E) Representative images of single myofiber sarcomeres from intact, contracted and cultured myofibers stained with α-actinin (Green) showing representative histograms of staining intensity and sarcomere spacing. F) Representative image of disorganized sarcomeres from injured myofibers stained with α-Actinin (Green) and MF20 (Red). G) Representative images and quantification of myofiber type from mouse Extensor digitorum longus and mouse Psoas muscle stained with Type 1 myofibers (Blue), Type 2a myofibers (Green), Type 2b myofibers (Red) and Wheat germ agglutinin (White). H) Representative image of human Psoas muscle cross sections stained with Laminin (Red) with I) quantification of average myofiber surface area and (J) myofiber surface area proportion from human Psoas myofibers compared to mouse Extensor digitorum longus and mouse psoas muscles using SMASH software. K) Representative image and quantification of mouse Extensor digitorum longus and mouse psoas myofiber lengths from isolated single myofibers. (K) Error bars represent mean ± SD, (G-J) Error bars represent mean ±SEM; (G, I-J) n = 3 biological replicates, (K) n = 40 myofibers per condition. **Figure S2:** Human satellite cells expand in situ, Related to Fig. 2. A) Quantification of average length of myofiber analyzed per experiment, whiskers represent min and max. B) Representative image of human myofibers showing centrally located nuclei stained with DAPI (Blue), Ki67 (Green), Pax7 (Red) and Dystrophin (White) and C) quantification of satellite cells per mm myofiber present at isolation on centrally nucleated fibers (CNF). D) Representative image of myofibers stained with DAPI (Blue), SDC4 (Green) Pax7 (Red) and Annexin-5 (White) with E) bisected myofibers serving as positive control stained for Annexin-5 (White) DAPI (Blue) and Pax7 (Red). F) Quantification of satellite cells expressing SDC4 at day 8 in culture. G) Representative image of satellite cells expressing M-Cadherin after isolation stained for DAPI (Blue), MCAD (Green) and Pax7 (Red). H) Representative image of satellite cell expansion on myofibers following 8 days in culture stained with DAPI (Blue), Ki67 (Green), Pax7 (Red) and Dystrophin (White) and quantification of I) Ki67 expression non-satellite cells per mm of myofiber, J) number of KI67 negative satellite cells per mm of myofiber and K) Ki67 expressing satellite cells per mm of myofiber across samples (s#). (A, C, K) Error bars represent mean ± SD, (F, I-K) Error bars represent mean ± SEM; (A) n = 351 myofibers. (C) n = averages from 20 (non-CNF) and 9 (CNF) myofibers. (F, I-K) n = 3 biological replicates. (K) n = averages from 4-22 myofibers, where individual data points represent individual myofibers. **Figure S3:** Myofiber culture unveils unique regenerative phenomena, Related to Fig. 3. Representative images of A) Representative image of cultured myofiber bundle stained for DAPI (Blue), MyoG (Green) and Pax7 (Red) (also presented in Figure 3A for reference). B) Representative image of myogenic progenitors and C) in situ de novo myofiber repair from fibers stained with DAPI (Blue), MyoG (Green) and MyoD (Red) where white dotted arrows outline the myocyte alignment. D) Representative images of cultured myofiber bundles stained for DAPI (Blue), pEGFR (Green) and Pax7 (Red). Quantification of E) total nuclei per mm of myofiber and across samples. F) Quantification of human satellite cells expressing Ki67 or Ki67 negative per mm of fiber across samples following culture in control or EGF containing media. G) Quantification proportion of nuclei expressing pax7 per myofiber. Quantification of H) proportion of satellite cells (Pax7+) stained negative for Ki67 and I) proportion of non-satellite cells (Pax7-) expressing Ki67 following culture in control or EGF containing media. J) quantification of MyoG-expressing nuclei per mm of myofiber across samples (s#). (E,F, J) Error bars represent mean ±SD, (E,G-I) Error bars represent means ± SD (EGF) and means ± SEM (Control); (E-I) n= 2 biological replicates EGF, 3 biological replicates control, (E, G, J) n = 4-32 myofibers, where individual data points represent individual myofibers. **Table S1:** Patient information used in this study. Patient information including sex, age, clinical complication, Psoas muscle mass, length and prefusion solution used during isolation. **Table S2:** Key resource Table.

## Data Availability

The datasets from the current study are available from the corresponding author on request.

## Abbreviations

DMD: Duchenne muscular dystrophy
ECM: Extracellular matrix
EDL: Extensor digitorum longus
EGF: Epidermal growth factor
EGFR: Epidermal growth factor receptor
FDA: Food and Drug Administration (USA)
MyHC: Myosin heavy chain
MyoG: Myogenin
