# Supplementary material for: Analysis of human satellite cell dynamics on cultured adult skeletal muscle myofibers
Source: Skelet Muscle. 2021 Jan 4;11:1. doi: 10.1186/s13395-020-00256-z (PMC7780694; doi:10.1186/s13395-020-00256-z)
Supplement: Supplementary file 1 — Additional file 1: Supplemental figures related to figures 1-3, patient information used in this study and key resource table. Figure S1: Myofibers from human Psoas muscle can be maintained in situ, Related to Fig. 1. A) Photographic overview of human Psoas minor myofiber bundle isolation showing expanded images of intact myofiber bundles (panel 9) and hypercontracted myofiber bundles (panel 10). Representative images of B) hypercontracted myofibers and C) myofibers with moderate damage stained for DAPI (Blue), α-Actinin (Green) and Myosin heavy chain (MF20, Red). D) Representative image of myofibers with minor damage stained for DAPI (Blue), Dystrophin (Green), Laminin (White) and IgG (Red). E) Representative images of single myofiber sarcomeres from intact, contracted and cultured myofibers stained with α-actinin (Green) showing representative histograms of staining intensity and sarcomere spacing. F) Representative image of disorganized sarcomeres from injured myofibers stained with α-Actinin (Green) and MF20 (Red). G) Representative images and quantification of myofiber type from mouse Extensor digitorum longus and mouse Psoas muscle stained with Type 1 myofibers (Blue), Type 2a myofibers (Green), Type 2b myofibers (Red) and Wheat germ agglutinin (White). H) Representative image of human Psoas muscle cross sections stained with Laminin (Red) with I) quantification of average myofiber surface area and (J) myofiber surface area proportion from human Psoas myofibers compared to mouse Extensor digitorum longus and mouse psoas muscles using SMASH software. K) Representative image and quantification of mouse Extensor digitorum longus and mouse psoas myofiber lengths from isolated single myofibers. (K) Error bars represent mean ± SD, (G-J) Error bars represent mean ±SEM; (G, I-J) n = 3 biological replicates, (K) n = 40 myofibers per condition. Figure S2: Human satellite cells expand in situ, Related to Fig. 2. A) Quantification of average length of myofiber analyzed pe [file 13395_2020_256_MOESM1_ESM.docx]

**SUPPLEMENTARY INFORMATION:**

**Analysis of Human Satellite Cell Dynamics on Cultured Adult Skeletal Muscle Myofibers**

Peter Feige, Eve C. Tsai, and Michael A. Rudnicki

**This PDF file includes:**

Supplemental legends and Figures S1 to S3

Supplemental Tables S1 and S2.

**
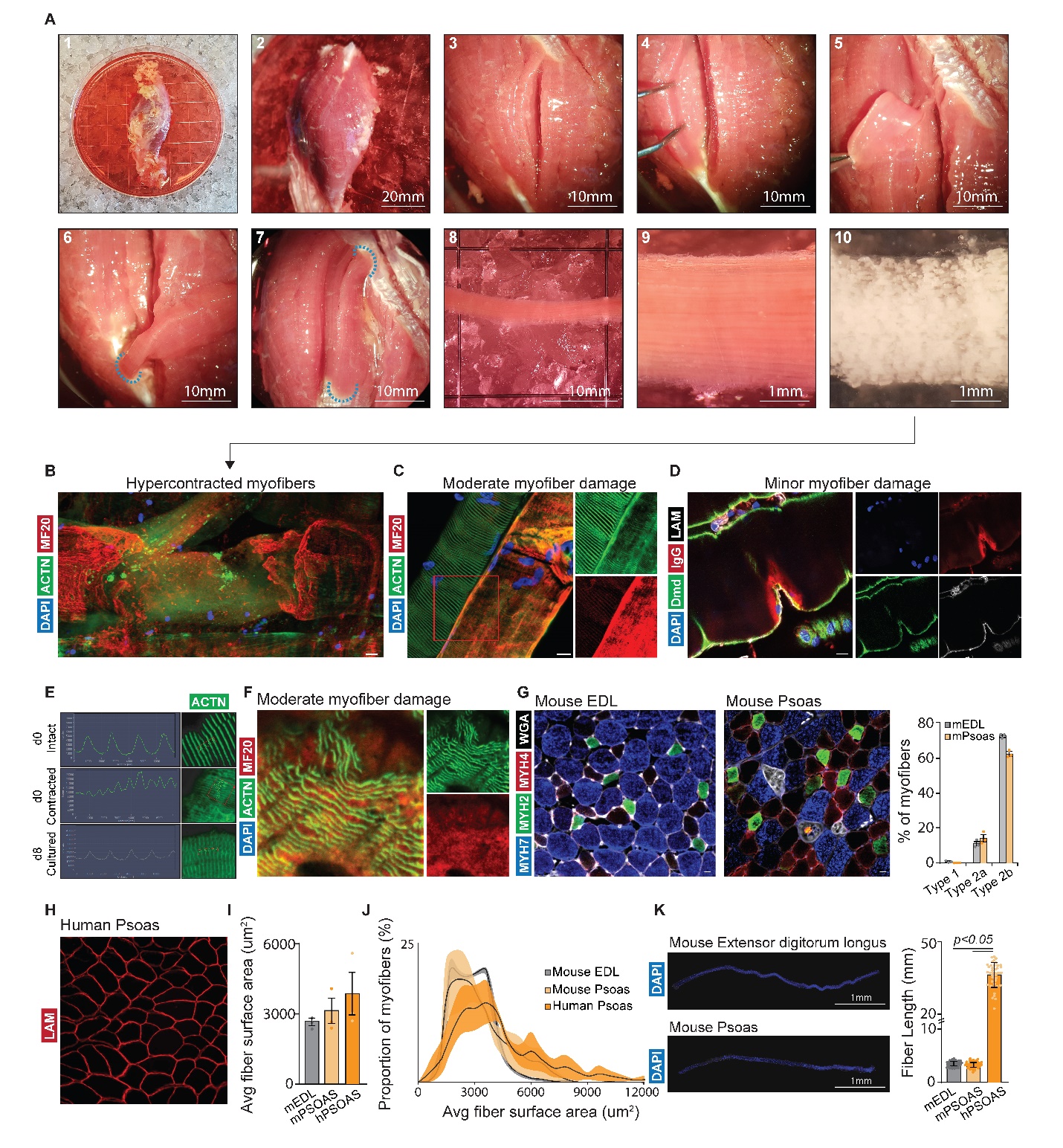
Figure S1: Myofibers from human Psoas muscle can be maintained *in situ*, Related to Figure 1.** A) Photographic overview of human Psoas minor myofiber bundle isolation showing expanded images of intact myofiber bundles (panel 9) and hypercontracted myofiber bundles (panel 10). Representative images of B) hypercontracted myofibers and C) myofibers with moderate damage stained for DAPI (Blue), α-Actinin (Green) and Myosin heavy chain (MF20, Red). D) Representative image of myofibers with minor damage stained for DAPI (Blue), Dystrophin (Green), Laminin (White) and IgG (Red). E) Representative images of single myofiber sarcomeres from intact, contracted and cultured myofibers stained with α-actinin (Green) showing representative histograms of staining intensity and sarcomere spacing. F) Representative image of disorganized sarcomeres from injured myofibers stained with α-Actinin (Green) and MF20 (Red). G) Representative images and quantification of myofiber type from mouse Extensor digitorum longus and mouse Psoas muscle stained with Type 1 myofibers (Blue), Type 2a myofibers (Green), Type 2b myofibers (Red) and Wheat germ agglutinin (White). H) Representative image of human Psoas muscle cross sections stained with Laminin (Red) with I) quantification of average myofiber surface area and (J) myofiber surface area proportion from human Psoas myofibers compared to mouse Extensor digitorum longus and mouse psoas muscles using SMASH software. K) Representative image and quantification of mouse Extensor digitorum longus and mouse psoas myofiber lengths from isolated single myofibers. (K) Error bars represent mean ± SD, (G-J) Error bars represent mean ±SEM; (G, I-J) n = 3 biological replicates, (K) n = 40 myofibers per condition.


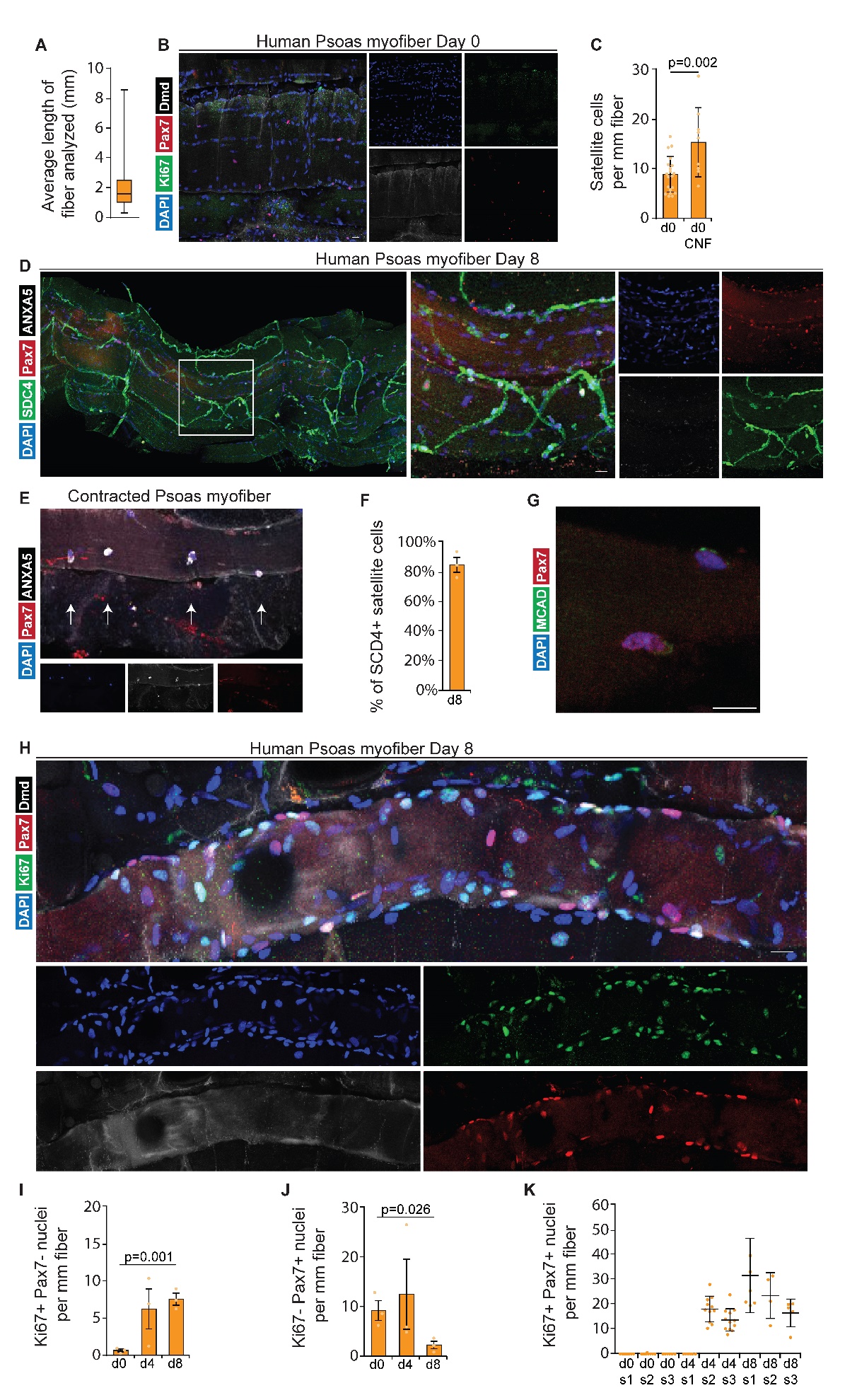


**Figure S2: Human satellite cells expand *in situ*, Related to Figure 2**. A) Quantification of average length of myofiber analyzed per experiment, whiskers represent min and max. B) Representative image of human myofibers showing centrally located nuclei stained with DAPI (Blue), Ki67 (Green), Pax7 (Red) and Dystrophin (White) and C) quantification of satellite cells per mm myofiber present at isolation on centrally nucleated fibers (CNF). D) Representative image of myofibers stained with DAPI (Blue), SDC4 (Green) Pax7 (Red) and Annexin-5 (White) with E) bisected myofibers serving as positive control stained for Annexin-5 (White) DAPI (Blue) and Pax7 (Red). F) Quantification of satellite cells expressing SDC4 at day 8 in culture. G) Representative image of satellite cells expressing M-Cadherin after isolation stained for DAPI (Blue), MCAD (Green) and Pax7 (Red). H) Representative image of satellite cell expansion on myofibers following 8 days in culture stained with DAPI (Blue), Ki67 (Green), Pax7 (Red) and Dystrophin (White) and quantification of I) Ki67 expression non-satellite cells per mm of myofiber, J) number of KI67 negative satellite cells per mm of myofiber and K) Ki67 expressing satellite cells per mm of myofiber across samples (s#). (A, C, K) Error bars represent mean ± SD, (F, I-K) Error bars represent mean ± SEM; (A) n = 351 myofibers. (C) n = averages from 20 (non-CNF) and 9 (CNF) myofibers. (F, I-K) n = 3 biological replicates. (K) n = averages from 4-22 myofibers, where individual data points represent individual myofibers.

**
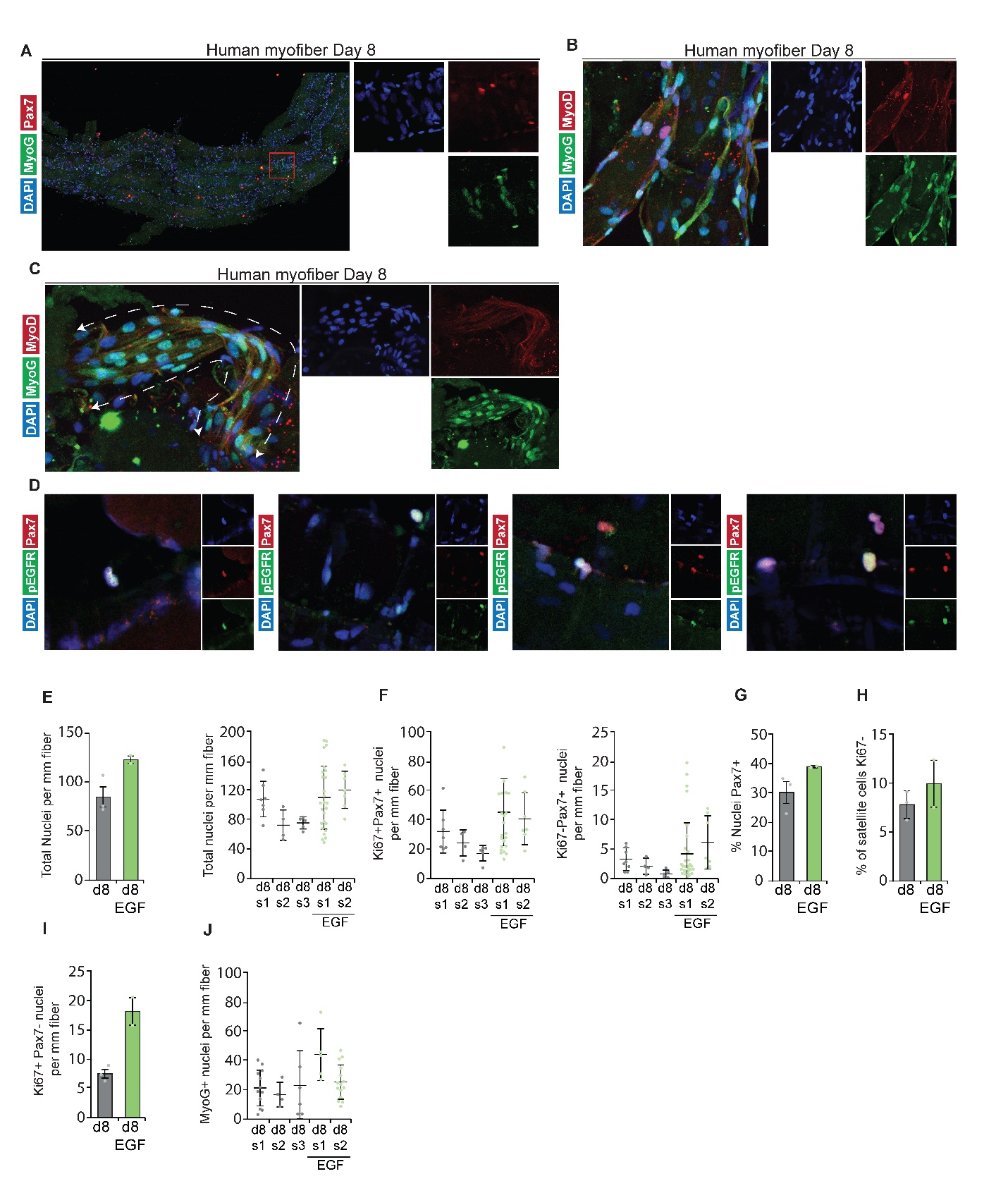
Figure S3: Myofiber culture unveils unique regenerative phenomena, Related to Figure 3.** Representative images of A) Representative image of cultured myofiber bundle stained for DAPI (Blue), MyoG (Green) and Pax7 (Red) (also presented in Figure 3A for reference). B) Representative image of myogenic progenitors and C) *in situ de novo* myofiber repair from fibers stained with DAPI (Blue), MyoG (Green) and MyoD (Red) where white dotted arrows outline the myocyte alignment. D) Representative images of cultured myofiber bundles stained for DAPI (Blue), pEGFR (Green) and Pax7 (Red). Quantification of E) total nuclei per mm of myofiber and across samples. F) Quantification of human satellite cells expressing Ki67 or Ki67 negative per mm of fiber across samples following culture in control or EGF containing media. G) Quantification proportion of nuclei expressing pax7 per myofiber. Quantification of H) proportion of satellite cells (Pax7+) stained negative for Ki67 and I) proportion of non-satellite cells (Pax7-) expressing Ki67 following culture in control or EGF containing media. J) quantification of MyoG-expressing nuclei per mm of myofiber across samples (s#). (E,F, J) Error bars represent mean ±SD, (E,G-I) Error bars represent means ± SD (EGF) and means ± SEM (Control); (E-I) n= 2 biological replicates EGF, 3 biological replicates control, (E, G, J) n = 4-32 myofibers, where individual data points represent individual myofibers.

**TABLE S1**

| Patient # | Sex | Age | Clinical complication | Psoas mass | Psoas length | Notes (perfusion solution) |
| --- | --- | --- | --- | --- | --- | --- |
| 1 | F | 68 | Subarachnoid hemorrhage anoxic brain injury | 22.10g | 10.95cm | Perfadex organ presentation solution |
| 2 | F | 64 | Subarachnoid hemorrhage | 12.06g | 9.55cm | Belzer UW cold storage solution |
| 3 | M | 50 | Unknown | 38.9g | 13.78cm | Custadiol HTK |

**Table S1: Patient information used in this study.** Patient information including sex, age, clinical complication, Psoas muscle mass, length and prefusion solution used during isolation.

**TABLE S2**

| REAGENT or RESOURCE | SOURCE | IDENTIFIER |
| --- | --- | --- |
| Antibodies | | |
| Mouse anti-Dystrophin | DSHB | Cat# MANEX1011B; RRID:AB_1157876 |
| Mouse anti-M-Cadherin | BD Biosciences | Cat# 611101, RRID:AB_398414 |
| Mouse anti-MHC | DSHB | Cat# MF 20; AB_2147781 |
| Mouse anti-MyH1 | DSHB | Cat# 6H1; AB_2314830 |
| Mouse anti-MyH2 | DSHB | Cat# SC-71; AB_2147165 |
| Mouse anti-MyH4 | DSHB | Cat# BF-F3; RRID: AB_2266724 |
| Mouse anti-MyH7 | DSHB | Cat# BA-F8; RRID: AB_10572253 |
| Mouse anti-MyoG | Novus | Cat# MAB66861; RRID:AB_10973343 |
| Mouse anti-Pax7 | DSHB | Cat# Ab528428; RRID: AB_528428 |
| Mouse anti-Syndecan-4 | Santa Cruz Biotechnology | Cat# sc-12766; RRID:AB_628314 |
| Mouse anti-α-Actinin | Sigma | Cat# A7732; RRID:AB_2221571 |
| Rabbit anti-Annexin-5 | Abcam | Cat# ab14196, RRID:AB_300979 |
| Rabbit anti-Ki67 | Abcam | Cat# ab15580; RRID:AB_443209 |
| Rabbit anti-MyoD | Abcam | Cat# ab133627 |
| Rabbit anti-p-EGFR | Cell Signaling technology | Cat# 3777S; RRID: AB_2096270 |
| Rat anti-Laminin | Sigma | Cat# L0663; RRID: AB_477153 |
| Rat anti-Perlecan | NSU Bioreagents | Cat# V2600; RRID:AB_2119238 |
| Wheat Germ Agglutinin Alexa 488 conjugate | Fisher | Cat# W11261 |
| Chemicals, Peptides and Recombinant Proteins | | |
| Chick Embryo Extract | MP Biomedicals | RRID 92850145 |
| Collagenase Type I | Worthington | Cat# LS004196 |
| Collagenase Type 2 | Worthington | Cat# LS004176 |
| Collagenase Type 3 | Worthington | Cat# LS004182 |
| Collagenase Type 4 | Worthington | Cat# LS004188 |
| Collagenase Type 5 | Worthington | Cat# LS005282 |
| Collagenase Type 6 | Worthington | Cat# LS005319 |
| Collagenase Type 7 | Worthington | Cat# LS005333 |
| Elastase | Worthington | Cat# LS006365 |
| Fibroblast Growth Factor-basic | EMD Millipore | Cat# GF003AFMG |
| ProClin950 | Sigma | Cat# 46879-U |
| Critical Commercial Assays | | |
| Human recombinant Epidermal Growth Factor | Miltenyi Biotech | Cat# 130-093-825 |
| TrueBlack | Biotium | Cat# 23007 |
| Software and Algorithms |  |  |
| GraphPad Prism | GraphPad Software Inc | https://www.graphpad.com/scientiﬁc-software/prism/ |
| ImageJ | NIH | https://imagej.nih.gov/ij/ |
| SMASH - semi-automatic muscle analysis  using segmentation of histology | Smith and Barton, 2014 | http://ﬁgshare.com/articles/SMASH_semi_automatic_muscle_analysis_using_segmentation_of_histology_a_MATLAB_application/1247634 |

**Table S2: Key resource Table.**

**CONTACT FOR REAGENT AND RESOURCE SHARING**

Information and requests for reagents may be directed to the Lead Contact, Michael A. Rudnicki (mrudnicki@ohri.ca).
